# Extended-interval dosing of rituximab/ocrelizumab is associated with a reduced decrease in IgG levels in multiple sclerosis

**DOI:** 10.1016/j.neurot.2025.e00554

**Published:** 2025-02-20

**Authors:** Camille Rigollet, Sean A. Freeman, Marine Perriguey, Jan-Patrick Stellmann, Lisa Graille-Avy, Jean-Christophe Lafontaine, Bruno Lemarchant, Tifanie Alberto, Sarah Demortière, Clémence Boutiere, Audrey Rico, Frédéric Hilézian, Pierre Durozard, Jean Pelletier, Adil Maarouf, Hélène Zéphir, Bertrand Audoin

**Affiliations:** a Aix Marseille Univ, APHM, Hôpital de la Timone, Marseille, France; b Department of Neurology, CRC-SEP, CHU of Lille, Lille, France; c Aix Marseille Univ, APHM, Hôpital de la Timone, Department of Neurology, Marseille, France; d Aix Marseille Univ, CNRS, CRMBM, Marseille, France; e APHM, Aix Marseille Univ, Hôpital de la Timone, Pôle d’Imagerie, CEMEREM, Marseille, France; f APHM, Aix Marseille Univ, Hôpital de la Timone, Department of Neuroradiology, Marseille, France; g Univ. Lille, INSERM, Laboratory of Neuroinflammation and Multiple Sclerosis (NEMESIS), U1172, Lille, France; h Centre Hospitalier d’Ajaccio, France

**Keywords:** Multiple sclerosis, Ocrelizumab, Rituximab, Safety, Hypogammaglobulinemia

## Abstract

The potential benefits of extended-interval dosing (EID) of rituximab (RTX) or ocrelizumab (OCR) in mitigating the reduction of immunoglobulin levels and decreasing the risk of infection in persons with relapsing-remitting multiple sclerosis (pwRRMS) remain largely unknown. We retrospectively analyzed two structured data collections including pwRRMS who were prescribed RTX/OCR using different interval dosing regimens, a 6-month standard-interval dosing (SD) or EID. The SD and EID cohorts included 88 and 271 pwRRMS, respectively, with a mean (SD) treatment duration of 3.5 (1.3) and 4.4 (1.5) years, and a mean (SD) interval between infusions of 6.4 (1.7) and 19.2 (11.9) months. After RTX/OCR initiation, the two cohorts did not differ in time to first relapse (*p* ​= ​0.83), time to first sustained accumulation of disability (*p* ​= ​0.98) and incidence of MRI activity (*p* ​= ​0.91). The time to first severe infectious event (SIE) was shorter in the SD cohort (*p* ​= ​0.005). The effect of treatment duration on reduction of serum IgG level was lower in the EID cohort (Estimate ​= ​0.15 ​g/L per year of follow-up, 95 ​% CI -0.06, −0.23, *p* ​= ​0.001). In the entire patient group, higher serum IgG levels at the last infusion were associated with a lower risk of SIE between two visits (HR ​= ​0.77 per g/L of serum IgG; 95 ​% CI: 0.66–0.91; *p* ​= ​0.006). This study suggests that EID of RTX/OCR may reduce the risk of serum IgG decline in pwRRMS without a loss of efficacy and may mitigate the risk of severe infections. These results must be confirmed by future randomized studies.

## Introduction

B-cell depleting therapies, including rituximab (RTX) and ocrelizumab (OCR), are associated with the highest risk of infections among all disease-modifying therapies (DMT) for relapsing remitting multiple sclerosis (RRMS) [1,2]. Furthermore, treatment with RTX/OCR has been associated with the highest risk of severe COVID-19 in patients with RRMS [[3], [4], [5]].

RTX/OCR-induced hypogammaglobulinemia could be one of the factors contributing to the increased risk of infection associated with RTX/OCR. Indeed, several studies evidenced that RTX/OCR therapies administered every 6 months in patients with MS are frequently associated with a decrease in IgG levels with or without hypogammaglobulinemia, and suggested that the IgG level could be associated with the risk of infection [[6], [7], [8], [9], [10], [11]].

Recent studies reported that the efficacy of RTX/OCR in RRMS can be maintained even if dosing intervals are extended beyond 6 months – a concept allowing a significant B-cell repopulation between infusions [[12], [13], [14], [15], [16], [17], [18], [19], [20], [21], [22], [23]]. A recent study performed in a limited sample of patients with RRMS followed during 12 months observed that EID of OCR (mean interval of 46 weeks) was associated with no serum IgG decrease contrary to a 6-month SD that was associated with significant decrease in IgG levels [24].

In this present study, we aim to determine the potential effect of EID of RTX/OCR in mitigating the risk of serum IgG decrease. As such, we performed a retrospective analysis of two structured data collections including patients with RRMS who were prescribed RTX/OCR in two French MS centers using either SD or EID.

## Methods

### Protocol and participants

We performed a retrospective analysis of two structured data collections, including patients with RRMS who were prescribed RTX/OCR in two expert French MS centers using two different dosing intervals. The MS center of Lille used until end of 2022 a 6-month SD with clinical evaluation every 6 months. As previously described, in 2018, the MS center in Marseille initiated a change in clinical practice concerning the dosing interval used for RTX/OCR in RRMS with the goal of improving safety [14]. All neurologists decided to propose to all patients an extension of the interval between two infusions to at least 12 months, maintaining clinical visits every 6 months. Generally, the EID was proposed directly after therapy induction or after the first re-infusion at 6 months. The 12-month minimum interval was based on the results of the pivotal phase II study of RTX in RRMS, demonstrating full maintenance of treatment efficacy at 12 months after infusion [25]. The timing for reinfusion after 12 months was chosen by the physician after discussing the risk-benefit assessment with the patient. Several factors were taken into account during the discussion, which included peripheral B-cell repopulation, disease activity before RTX/OCR onset, existence of residual disability, and any previous infections. Clinical visits were maintained every 6 months, and brain and spinal-cord MRI monitoring was performed at least annually for all patients.

Both centers used the same dosing regimen for RTX and OCR. For RTX, the dosing consisted of two 1 ​g doses administered two weeks apart for the first cycle, followed by 1 ​g for each subsequent cycle. For OCR, the dosing consisted of two 300 ​mg doses administered two weeks apart for the first cycle, followed by 600 ​mg for each subsequent cycle.

The two centers included patients who initiated RTX/OCR after January 2015 and had at least 24 months of follow-up since RTX/OCR initiation. We collected demographic information including age, sex, disease duration and previous DMTs. Patients were seen in both centers every 6 months for clinical evaluation. All examinations were performed by a neurologist of the department and severe infectious events (SIEs) were documented. SIEs were defined as a grade ≥3 according to the Common Terminology Criteria for Adverse Events v4.0 and were systematically reported in the medical chart. Serum IgG levels were measured before RTX/OCR initiation and at least every 6 months. The Expanded Disability Status Scale (EDSS) score was collected at each visit. All relapses since the last visit were recorded. All relapses since the last visit were recorded. Relapse was considered as the occurrence of neurological signs persisting >24 ​h, in the absence of fever, infection or other intercurrent phenomena.

### Ethical approval

The authors obtained ethical approval from their institutional review boards (approval no.: PADS-21-60 for Marseille and DEC21-347 for Lille).

### Statistical analysis

Sustained accumulation of disability (SAD) was evaluated according to the following definitions: increase in EDSS score by 1.5 points if the last EDSS score was 0, increase by 1 point if the EDSS score was 1–5.5, or increase by 0.5 points if the EDSS score was >5.5; confirmed after at least 6 months. Multivariate Cox proportional hazard models for recurrent events after RTX/OCR initiation were used to assess the risk of relapses, SAD and SIE after RTX/OCR initiation. To account for intraindividual correlation of observations, we included patient ID as a cluster variable. Longitudinal changes, e.g. serum IgG levels, were analyzed with linear mixed effect models. The Jonckheere-Terpstra test was used to explore the change of serum IgG levels over ordered groups of reinfusion intervals (0–6, 6–12, 12–18 and longer than 18 months). R v4.0.2, including the survival package, was used for statistical analysis, and *p* ​< ​0.05 was considered statistically significant.

The comparison of the proportions of patients with MRI activity, defined as at least one new T2 lesion or at least one enhancing lesion on brain or spinal cord MRI, was performed using Fisher's exact test.

### Data availability

All data analyzed during this study will be shared anonymized by reasonable request of a qualified investigator to the corresponding author.

## Results

### Study population

**Fig. 1:**
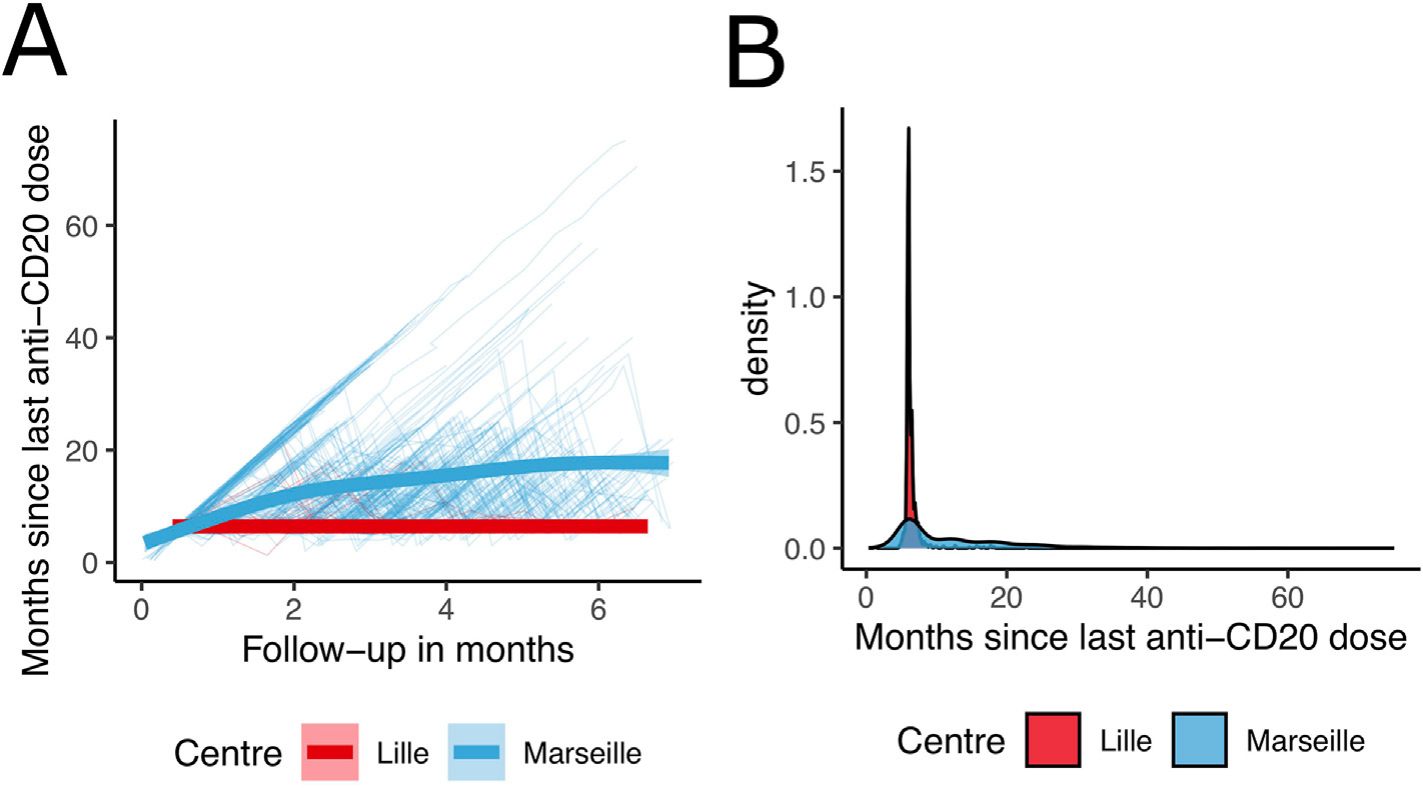
Treatment intervals in the two cohorts: 6-month standard-interval dosing cohort (Lille) and extended-interval dosing cohort (Marseille). (A) Change of time since last RTX/OCR infusion during the follow up; (B) Distribution of time since last RTX/OCR infusion at the two centers.

A total of 359 patients with RRMS treated with RTX/OCR were included: 88 in the MS center in Lille (SD cohort) and 271 in the MS center in Marseille (EID cohort) (eFigure1). The demographic and clinical characteristics of the patients are reported in Table 1. At RTX/OCR initiation, the mean (SD) age of patients was 37.7 (9.8) years for the SD cohort and 40.2 (12.1) years for the EID cohort. At RTX/OCR initiation, the mean (SD) disease duration was 8.2 (7.2) years for the SD cohort and 11.2 (8.1) years for the EID cohort. The median (range) EDSS at baseline was 2 (0–6.5) and 4 (0–8) for the SD cohort and EID cohort, respectively. The mean (SD) follow-up after RTX/OCR initiation was 3.5 (1.3) and 4.4 (1.5) years for the SD cohort and the EID cohort, respectively. During this time, the mean (SD) number of RTX/OCR cycles performed in the SD cohort and the EID cohort were, respectively, 7.6 (2.3) and 4.8 (2.6) corresponding to a mean (SD) interval between infusions of 6.4 (1.7) and 19.2 (11.9) months. In the EID cohort, the mean intervals between two infusions were shorter during the first two years of treatment and progressively increased thereafter (Fig. 1). The median number of 6-month standard-interval dosing was 2 (1–9) in the EID cohort before extension.

**Table 1:** Descriptive statistics of the two cohorts: 6-month standard-interval dosing (Lille) and extended-interval dosing (Marseille) of RTX/OCR.

	Lille cohort (SD) N ​= ​88	Marseille cohort (EID) N ​= ​271
**Sex,** F/M	55 (62.5 ​%)/33 (37.5 ​%)	193 (71.2 ​%)/78 (28.8 ​%)
**Age,** mean (SD); *years*	37.7 (9.8)	40.2 (12.1)
**Disease duration,** mean (SD); *years*	8.2 (7.2)	11.2 (8.1)
**DMT prior to RTX/OCR**	FING: 24 (27.3 ​%), NTZ: 9 (10.2 ​%); DMF: 12 (13.6 ​%); platform therapies: 10 (11.4 ​%); TRF: 14 (15.9 ​%); other: 1 (1.1 ​%), no DMT: 18 (20.5 ​%)	FING: 106 (39.1 ​%); NTZ: 81 (29.9 ​%); DMF: 7 (2.6 ​%); platform therapies: 12 (4.4 ​%); TRF: 11 (4.1 ​%); other: 24 (8.9 ​%); no DMT: 30 (11.1 ​%)
**Number of patients treated with RTX/OCR**	4/84	211/60
**EDSS at RTX/OCR initiation**; median [range]	2 [0–6.5]	4 [0–8]
**Serum IgG level at RTX/OCR initiation,** mean (SD); *gram***/***liter*	10.4 (2.8)	9.1 (2.1)
**Follow-up after RTX/OCR initiation,** mean (SD); *years*	3.5 (1.3)	4.4 (1.5)
**Interval between infusions of RTX/OCR,** mean (SD); *months*	6.4 (1.7)	19.2 (11.9)
**Number of RTX/OCR cycles since initiation,** median [range]	7 [4–14]	5 [1–15]

SD: standard-interval dosing; EID: extended-interval dosing; DMT: disease modifying therapy; FING: fingolimod; NTZ: natalizumab; DMF: dimethylfumarate; TRF: teriflunomid.

### MS clinical evolution during RTX/OCR in the two cohorts

**Fig. 2:**
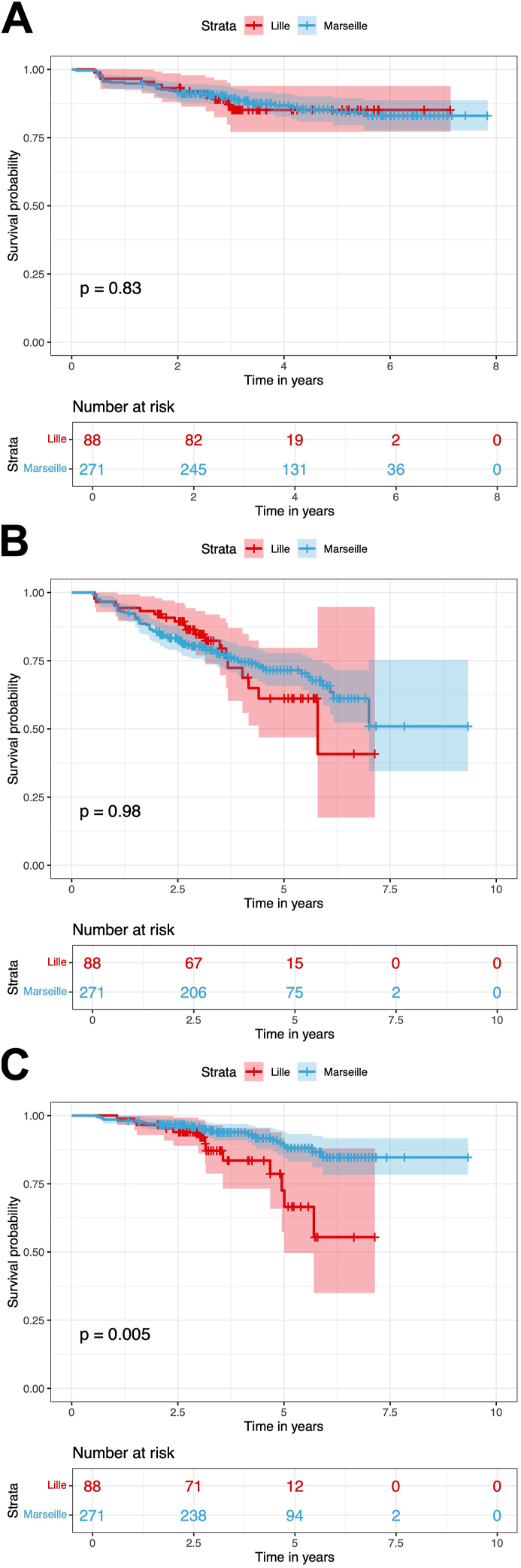
Kaplan-Meier Curve illustrating the time until the first relapse (A), the first sustained accumulation of disability event (B) and the first serious infectious event (C) after RTX/OCR initiation in the two cohorts: 6-month standard-interval dosing cohort (Lille) and extended-interval dosing cohort (Marseille). *p*-value from a log-rank test.

Analysis of the 2896 between visits intervals observed 53 visit intervals with relapses in 48 subjects (13.5 ​%). The two cohorts did not significantly differ with respect to the time to first relapse (*p* ​= ​0.83, Fig. 2). Cox model for recurrent relapse events including the delay since last RTX/OCR infusion, age, sex, EDSS score at last infusion, the number of previous RTX/OCR cycles and immunosuppressive DMT prior to RTX/OCR, indicated that the factors associated with a risk of relapse between two visits were the number of previous RTX/OCR cycles and age (Supplementary Table 1). The risk of relapse between two visits decreased with the increase in the number of previous RTX/OCR infusions (HR ​= ​0.71, 95 ​% CI 0.54, 0.93, *p* ​< ​0.012) and higher age (HR ​= ​0.97, 95 ​% CI 0.97–1.00, *p* ​< ​0.045) (Supplementary Table 1).

SAD events were observed for 129 of 2896 between visit intervals in 94 subjects (26 ​%). There was no difference in survival curve estimates between the two cohorts with respect to the time to first SAD (*p* ​= ​0.98, Fig. 2). Cox model for recurrent SAD events included the delay since last RTX/OCR infusion, age, sex, EDSS at last RTX/OCR infusion, the number of previous RTX/OCR infusions and immunosuppressive DMT prior to RTX/OCR. The factors associated with a risk of SAD between two visits were the number of previous RTX/OCR cycles, the delay since last RTX/OCR infusion and the EDSS at last RTX/OCR infusion (Supplementary Table 2). The risk of SAD between two visits decreased with the number of previous RTX/OCR cycles (HR ​= ​0.72, 95 ​% CI 0.60, 0.86, *p* ​< ​0.001) and the delay since last RTX/OCR infusion (HR ​= ​0.97, 95 ​% CI 0.95, 0.99, *p* ​< ​0.01) (Supplementary Table 2). Of note the delay since last RTX/OCR infusion increased in the EID cohort with the time since RTX/OCR initiation (Fig. 1) which could explain the inverse association found between risk of SAD and delay since last infusion. The risk of SAD between two visits increased with higher EDSS at last RTX/OCR infusion (HR ​= ​1.22, 95 ​% CI 1.11, 1.35, *p* ​< ​0.001) (Supplementary Table 2).

### MRI activity during RTX/OCR in the two cohorts

After the initiation of RTX/OCR treatment, the mean (SD) number of MRI scans per year was 1.26 (0.22) and 1.22 (0.41) for the SD and EID cohorts, respectively, totaling 381 and 1431 MRI scans over 310 and 1252 patient-years. Excluding re-baseline MRIs performed after RTX/OCR initiation, 13 and 40 scans identified new T2 lesions or enhancing lesions in the SD and EID cohorts, corresponding to rates of 4.19 and 3.19 per 100 patient-years, respectively (*p* ​= ​0.91).

### Evolution of IgG levels during RTX/OCR in the two cohorts

**Fig. 3:**
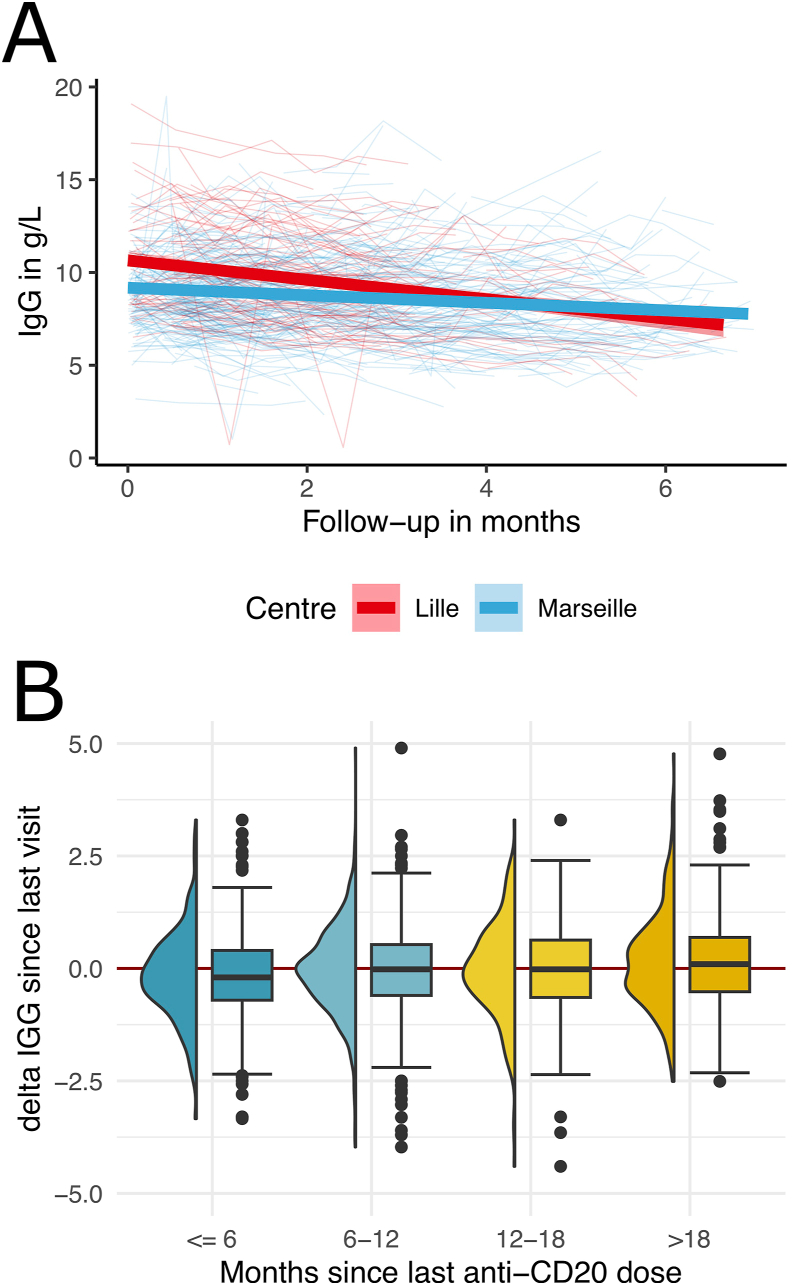
Serum IgG level in the two cohorts: 6-month standard-interval dosing cohort (Lille) and extended-interval dosing cohort (Marseille). (A) Serum IgG level evolution during the follow-up, (B) change in serum IgG levels between two visits as a grouped by the time since last RTX/OCR infusion.

At RTX/OCR initiation, the mean serum IgG level was lower in the EID cohort compared to the SD cohort (Fig. 1). At the end of the follow-up (mean (SD) 3.5 (1.3) years for the SD cohort and 4.4 (1.5) years for the EID cohort), the mean serum IgG level was higher in the EID cohort compared to the SD cohort (Fig. 3). During follow-up, serum IgG levels decreased with respect to the delay since RTX/OCR initiation (Estimate ​= ​−0.33 per year of treatment, 95 ​% CI -0.40, −0.27, <0.001), higher age (Estimate ​= ​−0.02 per year, 95 ​% CI -0.02, −0.01, *p* ​< ​0.001) and were lower in males (Estimate ​= ​−0.36, 95 ​% CI -0.58, −0.14, *p* ​= ​0.002). The interaction between center and time since RTX/OCR initiation evidenced that the effect of treatment duration on serum IgG level was lower in the EID cohort than the SD cohort (Estimate ​= ​0.15 ​G/L per year of follow-up, 95 ​% CI -0.06, −0.23, *p* ​= ​0.001) (Fig. 3 and Table 2). We further analyzed potential factors associated with the level of serum IgG change between two consecutive visits. The model included the delay since last RTX/OCR infusion, age, sex, IgG level at RTX/OCR initiation and immunosuppressive DMT prior to RTX/OCR. The factors associated with change of serum IgG level since last RTX/OCR infusion were the serum IgG level at RTX/OCR initiation and the time since last RTX/OCR infusion (Table 3). Higher serum IgG levels at the time of RTX/OCR initiation were associated with a higher serum IgG level decrease (Estimate ​= ​−0.12 ​G/L per G, 95 ​% CI -0.14, −0.10, *p* ​< ​0.001). In contrast, longer time since last RTX/OCR infusion was associated with a less pronounced decrease of serum IgG levels since last RTX/OCR infusion (Estimate ​= ​0.01 ​G/L per month, 95 ​% CI 0.00, 0.02, *p* ​= ​0.003). Analysis of the mean IgG changes since last RTX/OCR infusion according to the different time since last infusion (807 intervals ≤6 months; 1190 intervals >6 months and ≤12 months; 382 intervals >12 months and ≤18 months and 517 intervals >18 months) observed a decrease of mean IgG level only for intervals ≤6 months (Fig. 2B). The Jonckheere-Terpstra test showed lower IgG decrease with increasing delay (*p* ​< ​0.001).

**Table 2:** Linear mixed effect model for serum IgG level during RTX/OCR in the two cohorts.

*Predictors*	Serum IgG level		
	*Estimates*	*CI*	*p*
(Intercept)	11.21	10.66–11.76	<0.001
Centre [EID cohort]	−1.32	−1.81–−0.84	<0.001
Delay since RTX/OCR initiation	−0.33	−0.40–−0.27	<0.001
Age	−0.02	−0.02–−0.01	<0.001
SEX [M]	−0.36	−0.58–−0.14	0.002
Centre [EID cohort] ​× ​delay since RTX/OCR initiation	0.15	0.06–0.23	0.001

**Table 3:** Linear mixed effect model for change in serum IgG level between two visits during RTX/OCR in the two cohorts.

*Predictors*	Change in serum IgG level between two visits		
	*Estimates*	*CI*	*p*
(Intercept)	1.10	0.76–1.44	<0.001
Serum IgG level at RTX/OCR initiation	−0.12	−0.14–−0.10	<0.001
Delay since last RTX/OCR infusion	0.01	0.00–0.02	0.003
Age	−0.00	−0.01–0.00	0.398
Sex [M]	−0.05	−0.18–0.07	0.408
Immunosuppressive (IS) DMT prior to RTX/OCR initiation	−0.08	−0.23–0.06	0.255

DMT: disease modifying therapy; immunosuppressive DMT included all DMTs for multiple sclerosis except platform therapies.

### Frequency of SIEs during RTX/OCR in the two cohorts

We observed 46 SIEs in 37 subjects (10.5 ​%) (Supplementary Table 3). SIEs are mostly represented by lower respiratory tract infections, with 26 (56.5 ​%) events including 12 (26 ​%) severe COVID-19 which led to death in one patient, 12 (26 ​%) pneumonia cases and 2 (4.5 ​%) severe influenza cases. Upper urinary tract infections were the second most frequent SIEs with 5 (11 ​%) events. The two cohorts significantly differed concerning their survival curve estimates with respect to time to first SIE after RTX/OCR initiation with a shorter time to first SIE in the SD cohort (*p* ​= ​0.005) (Fig. 2).

The evolution of COVID-19 infection incidence in the general population was highly similar between the two regions (eFigure 2). Most patients in both cohorts were exposed to the COVID-19 pandemic after the initiation of treatment (eFigure 3). A comparison of the evolution of SIE incidence between the two cohorts revealed a higher incidence in the SD cohort during the COVID-19 pandemic (eFigure 4).

The Cox model for SIEs including the delay since last RTX/OCR infusion, age at last RTX/OCR infusion, sex, EDSS score at last RTX/OCR infusion, the serum IgG level at last RTX/OCR infusion and immunosuppressive DMT prior to RTX/OCR initiation, evidenced that the serum IgG level at last RTX/OCR infusion was the only factor associated with the risk of SIE between two visits (Table 4). Higher serum IgG level at last RTX/OCR infusion was associated with lower risk of SIE between two visits (HR ​= ​0.77 ​G/L of serum IgG, 95 ​% CI 0.66, 0.91, *p* ​= ​0.006) (Table 4). Median (range) value of the last serum IgG level before SIE was 6.8 ​G/L (4.7–14.1).

**Table 4:** Multivariate Cox proportional hazard models for recurrent serious infectious events in the two cohorts.

	Beta (SE)	HR (95 ​% CI)	p
Delay since last RTX/OCR infusion (per month)	−0.01 (0.02)	0.99 (0.96, 1.03)	0.705
Age at last RTX/OCR infusion (per year)	0.00 (0.02)	1.00 (0.97, 1.04)	0.844
SEX			
F (ref)	–	–	–
M	0.33 (0.34)	1.38 (0.71, 2.70)	0.340
Serum IgG level at last RTX/OCR infusion (per gram/liter)	−0.26 (0.08)	0.77 (0.66, 0.91)	0.002
EDSS score at last RTX/OCR infusion	0.07 (0.10)	1.08 (0.89, 1.30)	0.454
Immunosuppressive (IS) DMT prior to RTX/OCR initiation			
N (ref)	–	–	–
IS	1.12 (0.73)	3.06 (0.72, 12.88)	0.128

DMT: disease modifying therapy; immunosuppressive DMT included all DMT for multiple sclerosis except platform therapies.

## Discussion

The present study suggests that EID of RTX/OCR in patients with RRMS was associated with lower risk of serum IgG decrease and that EID may mitigate the risk of serious infections. Crucially, the present study provides further data arguing that EID of RTX/OCR is not associated with a higher clinical or radiological disease activity or disability progression in patients with RRMS compared to SD.

Several recent studies have observed that EID of RTX/OCR in patients with RRMS is not associated with increased disease activity despite significant B-cell repopulation between two infusions [[12], [13], [14], [15], [16], [17], [18], [19], [20], [21], [22], [23]]. Accordingly, we did not observe in the present study higher risk of relapse, MRI activity or disability progression in patients with a mean dosing interval greater than 12 months, compared to patients treated with a standard 6-month dosing interval. These findings suggest that RTX/OCR may act as an induction therapy, and that continuous deep B-cell depletion by maintenance therapy is not necessary to achieve efficacy of RTX/OCR in RRMS. Importantly, we also did not observe clinical disease reactivation in patients treated with extended dosing just after their first treatment cycle providing another argument for an induction effect of RTX/OCR in MS.

As previously described, the present study demonstrates that RTX/OCR are associated with a progressive decrease in serum IgG levels [11,26,27]. During follow-up, we observed that serum IgG levels significantly decreased over time from the initiation of RTX/OCR, with a mean decrease of −0.33 ​g/L per year of treatment. Importantly, this study included patients with two different dosing interval regimens, allowing for the assessment of the potential impact of the time interval between consecutive infusions on the decline in serum IgG levels. An analysis of 2896 intervals showed that a longer time since the last RTX/OCR infusion was associated with a less pronounced decrease in serum IgG levels. We observed that serum IgG levels only decreased when the time since the last infusion was approximately 6 months. When the interval exceeded 6 months, mean serum IgG levels remained stable or increased for intervals longer than 18 months. This finding has important clinical implications, suggesting that even a limited EID of RTX/OCR may provide a safety benefit. Finally, we identified two non-modifiable factors associated with a higher risk of serum IgG decline: baseline IgG levels at the first RTX/OCR infusion and male sex.

In the present cohorts of patients treated with RTX/OCR over a mean period of 4 years, we observed 46 SIEs, corresponding to a crude incidence of 2.5 and 4.8 per 100 patient-years for the EID and SD cohorts, respectively. As previously reported in patients treated with B-cell depleting therapies, SIEs are mostly represented by lower respiratory tract infections, with 26 (56.5 ​%) events including 12 (26 ​%) severe COVID-19 which led to death in one patient, 12 (26 ​%) pneumonia cases and 2 (4.5 ​%) severe influenza cases. Upper urinary tract infections were the second most frequent SIEs with 5 (11 ​%) events. When comparing the two cohorts treated with different regimens (SD versus EID), we found that the time to the first SIE was significantly shorter in patients treated with the SD regimen. The SD group exhibited a higher risk of SIEs, despite being younger, having a lower EDSS score, and higher serum IgG levels at RTX/OCR initiation. Of note, we observed a particularly high rate of SIE in the SD cohort compared to previously published data. This difference is likely attributable to the COVID-19 pandemic, which began after treatment initiation for most patients in both cohorts. Notably, the rates of SIE following the onset of the COVID-19 pandemic differed significantly between the two cohorts, suggesting that the EID scheme is associated with a lower rate of severe COVID-19. The pandemic may have amplified the infection risk mitigation benefits of the EID approach.

Crucially, the SD cohort experienced a faster decline in serum IgG levels throughout the follow-up compared to the EID cohort. This accelerated decrease in IgG levels associated with the SD regimen could partly explain the higher risk of SIEs related to this dosing scheme. Notably, the divergence in the survival curves for the first SIE between the two cohorts (occurring after 4 years of RTX/OCR) coincided with the point at which the mean serum IgG levels in the SD cohort dropped below those of the EID cohort. However, due to the limited number of SIE events and the relatively small size of the SD cohort, the comparison of SIE risk between the two groups should be interpreted with caution and should be confirmed by further studies.

Interestingly, when we examined predictors of serious infections in the entire cohort, we found that serum IgG levels were the strongest explanatory factor. The association between serum IgG levels and infection risk has been previously reported in patients with MS treated with RTX/OCR [[6], [7], [8], [9], [10], [11]]. Strikingly, we found an association with the absolute value of serum IgG level prior to SIE, suggesting that any decrease of serum IgG level impacts the risk of SIE by reducing the patient's level of protection. These preliminary results should be confirmed by further studies with larger sample sizes. If replicated, they could have significant clinical implications, as they suggest that any factors leading to a decrease in serum IgG levels in a patient could potentially increase their risk of SIE. However, even if any decrease of IgG level could impact the risk of SIE, it is important to note that half of the SIEs occurred in patients with an IgG level below 7 ​G/L, arguing that hypogammaglobulinemia may be a determining factor.

We did not observe an association between higher EDSS score and risk of infection, as previously reported in numerous studies [6,10]. Notably, we included in the present study only patients with RRMS, with a mean age below 40 years and low disability (mean EDSS lower than 4). These characteristics presumably explain the lack of association found here between EDSS and risk of SIEs, mostly driven by the high frequency of swallowing and urinary tract dysfunctions in patients with higher EDSS scores included in previous studies.

The present study has several limitations. First, we cannot exclude differences between the two centers in terms of data collection for SIEs. Indeed, although patient monitoring—including clinical visits, IgG measurements, and MRI exams—was standardized and predefined, both data extraction and the analysis plan were conducted retrospectively. However, we expect limited difference of SIE data collection between the two centers given the severity of these events, which are usually reported systematically. Moreover, the higher decrease of serum IgG level observed in the group treated with the SD and the association found between serum IgG level and risk of SIEs argue for a direct impact of the two dosing regimens rather than a bias related to center effects. Secondly, the sample sizes were limited especially for the SD cohort.

The present observational study suggests a safety benefit of EID of RTX/OCR in patients with RRMS, as it significantly reduces the risk of IgG decline and potentially the risk of SIEs. These findings should be confirmed by future randomized clinical trials.

## Author contributions

Camille Rigollet played a major role in the acquisition of the data, gathering of data and writing the manuscript.

Sean A. Freeman played a major role in the acquisition of the data, gathering of data and writing the manuscript.

Marine Perriguey played a major role in the acquisition of the data, gathering of data and writing the manuscript.

Jan-Patrick Stellmann played a major role in conducting statistical analysis.

Lisa Graille-Avy played a major role in the acquisition of the data and the gathering of data.

Jean-Christophe Lafontaine played a major role in the acquisition of the data.

Bruno Lemarchant played a major role in the acquisition of the data.

Tifanie Alberto played a major role in the acquisition of the data.

Sarah Demortière played a major role in the acquisition of the data.

Clémence Boutiere played a major role in the acquisition of the data.

Audrey Rico played a major role in the acquisition of the data.

Frédéric Hilézian played a major role in the acquisition of the data.

Pierre Durozard played a major role in the acquisition of the data.

Jean Pelletier played a major role in the acquisition of the data.

Adil Maarouf played a major role in the acquisition of the data.

Hélène Zéphir planned and conducted the study, and wrote the manuscript.

Bertrand Audoin planned and conducted the study, and wrote the manuscript.

## Funding/Support

This observational study was not supported by any specific funding.

## Declaration of competing interest

The authors declare that they have no known competing financial interests or personal relationships that could have appeared to influence the work reported in this paper.
