# Supplementary material for: Extended-interval dosing of rituximab/ocrelizumab is associated with a reduced decrease in IgG levels in multiple sclerosis
Source: Neurotherapeutics. 2025 Feb 20;22(3):e00554. doi: 10.1016/j.neurot.2025.e00554 (PMC12047468; doi:10.1016/j.neurot.2025.e00554)
Supplement: Multimedia component 1 [file mmc1.pdf]

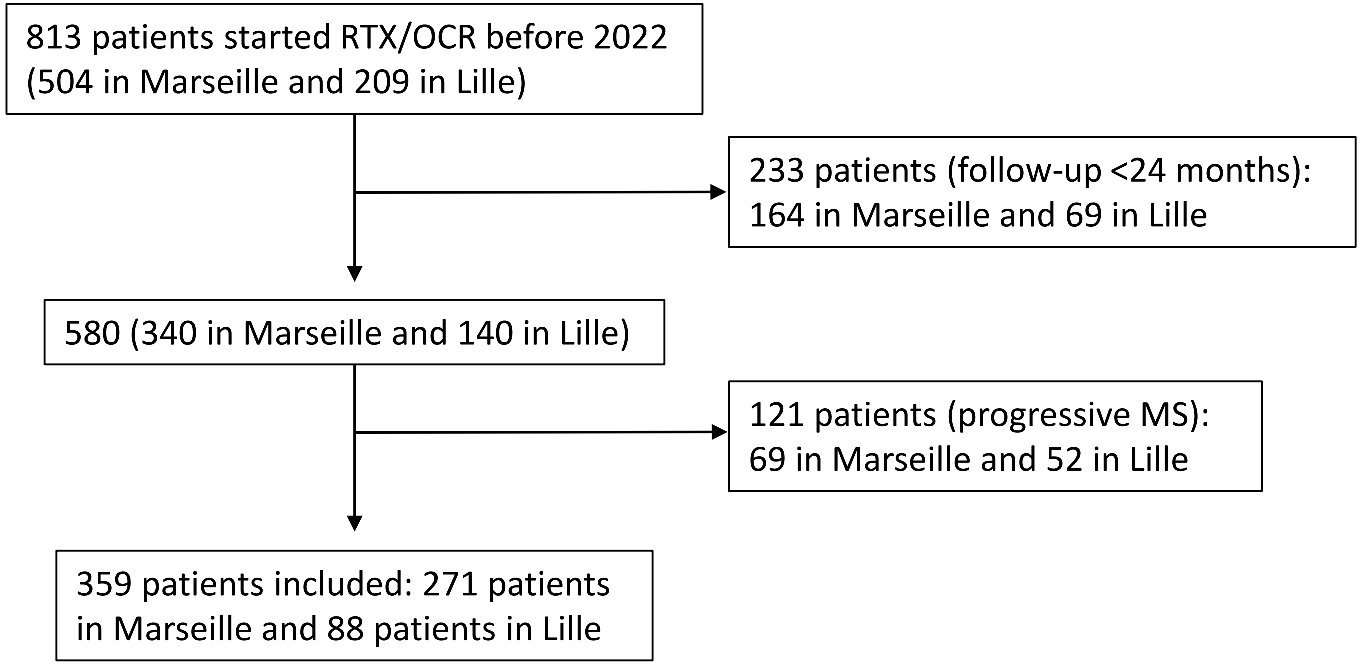
813 patients started RTX/OCR before 2022 (504 in Marseille and 209 in Lille)
233 patients (follow-up <24 months): 164 in Marseille and 69 in Lille
580 (340 in Marseille and 140 in Lille)
121 patients (progressive MS): 69 in Marseille and 52 in Lille
359 patients included: 271 patients in Marseille and 88 patients in Lille
