# Supplementary figures and images for: Extended-interval dosing of rituximab/ocrelizumab is associated with a reduced decrease in IgG levels in multiple sclerosis
Source: Neurotherapeutics. 2025 Feb 20;22(3):e00554. doi: 10.1016/j.neurot.2025.e00554 (PMC12047468; doi:10.1016/j.neurot.2025.e00554)

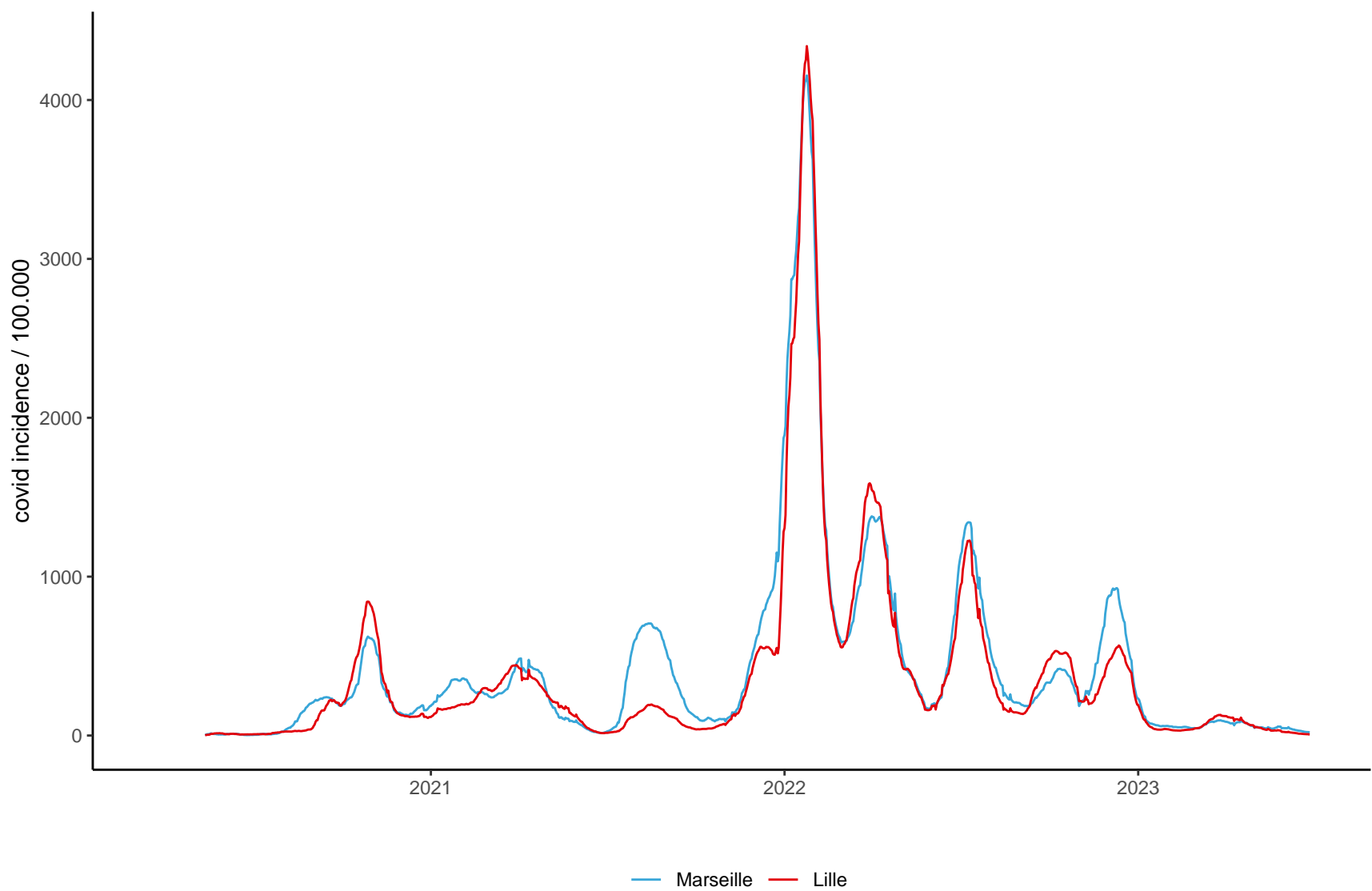

Supplement: Multimedia component 2 [file mmc2.pdf]

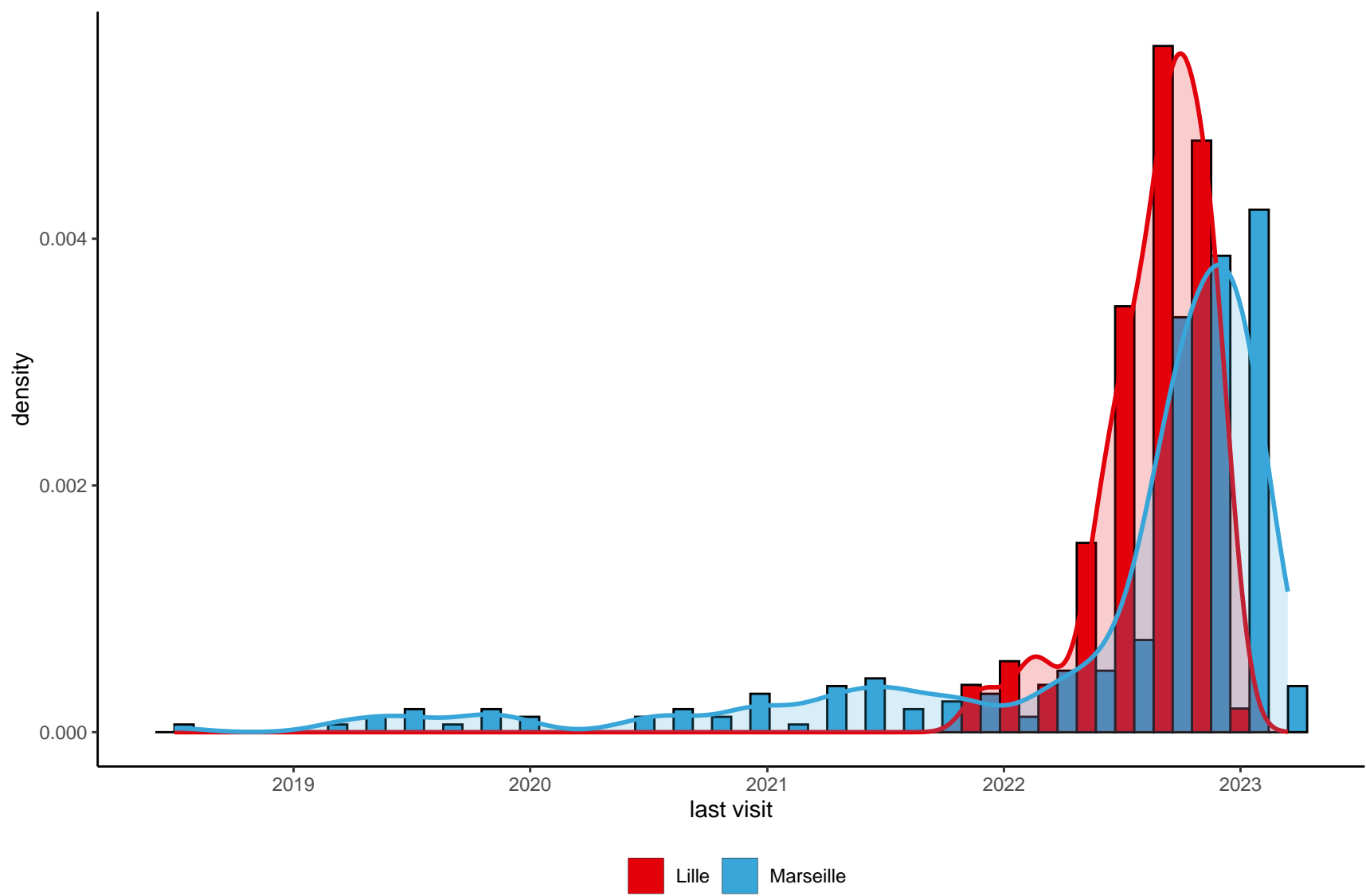

Supplement: Multimedia component 3 [file mmc3.pdf]

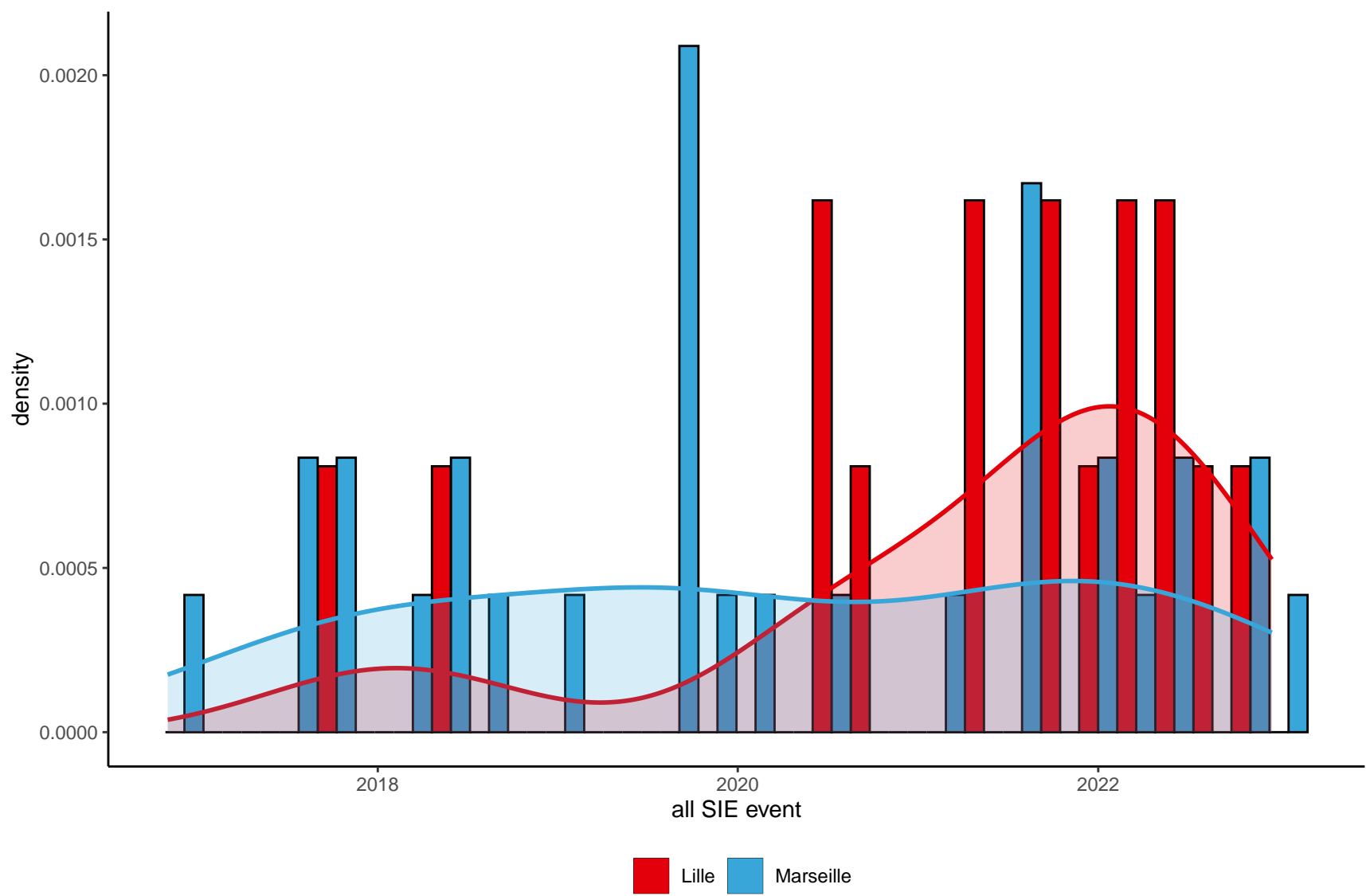

Supplement: Multimedia component 4 [file mmc4.pdf]
