# Supplementary material for: Extended-interval dosing of rituximab/ocrelizumab is associated with a reduced decrease in IgG levels in multiple sclerosis
Source: Neurotherapeutics. 2025 Feb 20;22(3):e00554. doi: 10.1016/j.neurot.2025.e00554 (PMC12047468; doi:10.1016/j.neurot.2025.e00554)
Supplement: Multimedia component 5 [file mmc5.docx]

**Supplementary Table 1. Multivariate Cox proportional hazard models for recurrent relapse events in the two cohorts**

| *Variable* | *Beta (SE)* | *HR (95% CI)* | *p* |
| --- | --- | --- | --- |
| Delay since last RTX/OCR infusion (per month) | -0.03 (0.03) | 0.97 (0.93, 1.02) | 0.268 |
| Age | -0.03 (0.02) | 0.97 (0.95, 1.00) | 0.045 |
| Sex |  |  |  |
| F (ref) | – | – | – |
| M | 0.31 (0.31) | 1.36 (0.76, 2.43) | 0.297 |
| EDSS score at last RTX/OCR infusion | 0.06 (0.08) | 1.06 (0.90, 1.25) | 0.471 |
| Number of previous RTX/OCR cycles | -0.34 (0.15) | 0.71 (0.54, 0.93) | 0.012 |
| Immunosuppressive (IS) DMT prior to RTX/OCR initiation |  |  |  |
| N (ref) | – | – | – |
| IS | 0.57 (0.45) | 1.78 (0.74, 4.24) | 0.195 |

DMT: disease modifying therapy; immunosuppressive DMT included all DMTs for multiple sclerosis except platform therapies.

**Supplementary Table 2. Multivariate Cox proportional hazard models for recurrent sustained accumulation of disability events in the two cohorts**

| *Variable* | *Beta (SE)* | *HR (95% CI)* | *p* |
| --- | --- | --- | --- |
| Delay since last RTX/OCR infusion (per month) | -0.03 (0.01) | 0.97 (0.95, 0.99) | 0.010 |
| Age | 0.00 (0.01) | 1.00 (0.98, 1.02) | 0.922 |
| Sex |  |  |  |
| F (ref) | – | – | – |
| M | 0.13 (0.19) | 1.14 (0.77, 1.67) | 0.511 |
| EDSS score at last RTX/OCR infusion | 0.20 (0.05) | 1.22 (1.11, 1.35) | <0.001 |
| Number of previous RTX/OCR cycles | -0.33 (0.08) | 0.72 (0.60, 0.86) | <0.001 |
| Immunosuppressor (IS) DMT prior to RTX/OCR initiation |  |  |  |
| N (ref) | – | – | – |
| IS | -0.09 (0.24) | 0.91 (0.53, 1.56) | 0.735 |

DMT: disease modifying therapy; immunosuppressive DMT included all DMTs for multiple sclerosis except platform therapies.

**Supplementary Table 3. Number and type of serious infectious events during RTX/OCR in the two cohorts. Patient-years (PY)**

| **Type of SIE** | **SIE Cohort (1252**  **PY)** | **SD cohort (310 PY)** |
| --- | --- | --- |
| Severe COVID19 | 5 | 7 |
| Pneumonia | 9 | 3 |
| Upper urinary tract infection | 3 | 2 |
| Severe non localized infectious syndrome | 3 |  |
| Osteoarticular infection | 3 |  |
| Necrotizing throat involving soft tissue | 1 | 1 |
| Severe influenza | 2 |  |
| Abdominal infections | 1 | 1 |
| Severe skin infection | 1 |  |
| Disseminated zoster | 1 |  |
| Severe genital infection | 1 |  |
| Necrotizing dental infection | 1 |  |
| Viral myocarditis |  | 1 |
| **Total** | **31 (2.5 per 100 PY)** | **15 (4.8 per 100 PY)** |
